# Empagliflozin Attenuates High-Glucose-Induced Astrocyte Activation and Inflammation via NF-κB Pathway

**DOI:** 10.3390/cimb46110737

**Published:** 2024-11-04

**Authors:** Dong Hee Kim, Min Jin Lee, Dasol Kang, Ji Young Lee, Sujin Park, Ah Reum Khang, Ji Hyun Bae, Joo Yeon Kim, Su Hyun Kim, Yang Ho Kang, Dongwon Yi

**Affiliations:** 1 Research Institute for Convergence of Biomedical Science and Technology, Pusan National University Yangsan Hospital, Yangsan 50621, Republic of Korea; gainlydh@pusan.ac.kr (D.H.K.); minjinpig@hanmail.net (M.J.L.); lainef7@ulsan.ac.kr (D.K.); leejy2828@naver.com (J.Y.L.); sujini0807@gmail.com (S.P.); medikar82@gmail.com (A.R.K.); yaho1229@gmail.com (J.H.B.); rlawndus0226@naver.com (J.Y.K.); carpediem1113@gmail.com (S.H.K.); kangyh@pusan.ac.kr (Y.H.K.); 2 Division of Endocrinology and Metabolism, Department of Internal Medicine, Pusan National University Yangsan Hospital, Pusan National University School of Medicine, Yangsan 50621, Republic of Korea

**Keywords:** sodium-glucose cotransporter 2 inhibitor, diet, high-fat diet, hypothalamus, astrocyte, NF-κB

## Abstract

Sodium-glucose cotransporter-2 (SGLT2) inhibitors regulate blood glucose levels in patients with type 2 diabetes mellitus and may also exert anti-inflammatory and anti-atherosclerotic effects by promoting M2 macrophage polarization. Although SGLT2 is expressed in brain regions that influence glucose balance and cognitive function, its roles in the central nervous system are unclear. This study investigated the effects of empagliflozin (EMPA), an SGLT2 inhibitor, on hypothalamic inflammation associated with metabolic diseases. Mice were subjected to a high-fat diet (HFD) for varying durations (3 d, 3 weeks, and 16 weeks) and treated with EMPA for 3 weeks (NFD, NFD + EMPA, HFD, HFD + EMPA; *n* = 5/group). EMPA regulated the expression of astrocyte markers and pro-inflammatory cytokine mRNA in the hypothalamus of HFD-induced mice, which was linked to regulation of the NF-κB pathway. Under hyperglycemic conditions, EMPA may mitigate hypothalamic inflammation by modulating astrocyte activation via the NF-κB pathway. Our findings demonstrated that EMPA possesses therapeutic potential beyond merely lowering blood glucose levels, opening new avenues for addressing inflammation and providing neuroprotection in metabolic disease management.

## 1. Introduction

Obesity is caused by a disparity between dietary energy intake and energy expenditure through physical activity. A high-fat diet (HFD) is a risk factor for various diseases, including type 2 diabetes mellitus [1,2], hypertension [3,4], heart failure [5,6], and dyslipidemia [7]. Obesity impairs normal energy (glucose) utilization and lipolysis, causing an accumulation of circulating free fatty acids (FFAs) and pro-inflammatory cytokines, ultimately leading to inflammation [8,9].

The inflammation induced by obesity not only affects peripheral tissues but also triggers chronic inflammation in the central nervous system (CNS), which is associated with neurodegenerative diseases, such as Alzheimer’s disease [10,11], Parkinson’s disease [12], and multiple sclerosis [13]. The hypothalamus is a key structure in the CNS that serves various systemic metabolic functions by regulating essential physiological processes, including nutrient sensing [14,15], appetite control [16], energy expenditure [17], glucose homeostasis [18], and carbohydrate and lipid metabolism [19]. Notably, an HFD has been shown to increase inflammatory responses in the hypothalamus, promote insulin and leptin resistance, and disrupt energy homeostasis. These impairments prevent proper energy utilization, leading to irregular appetite control and the exacerbation of obesity [20,21,22].

Emerging evidence suggests that astrocytes and non-neuronal cells regulate body weight and metabolize nutrients. Astrocytes support neurons, modulate synaptic activity, maintain blood–brain barrier integrity, and regulate glucose transport into the brain. Increased levels of circulating FFAs entering the brain due to an HFD can promote astrocyte reactivation, lipid accumulation, and pro-inflammatory cytokine secretion [23,24], which may exacerbate obesity. The early stages of HFD consumption are associated with the proliferation, reactivation, and morphological transformation of astrocytes [25]. Activated astrocytes exert neurotoxic effects by producing pro-inflammatory cytokines, such as TNF-α and IL-1β [26], and regulating several inflammatory pathways, including the classic pro-inflammatory inhibitor of κB (IκBα) kinase β/nuclear factor kappa B (IKKβ/NF-κB) signaling pathway [27,28]. Notably, inhibiting the NF-κB pathway in astrocytes can reduce astrogliosis and mitigate HFD-induced weight gain [25], while also restoring hypothalamic leptin sensitivity [29]. This reflects the potential of NF-κB pathway-targeting therapies for obesity associated with HFD consumption.

Sodium-glucose cotransporter 2 (SGLT2) inhibitors, a class of antidiabetics, effectively lower blood glucose levels via an insulin-independent pathway [30,31]. Several types of SGLT2 inhibitors, including empagliflozin (EMPA), have attracted attention because of their ability both to regulate blood glucose by inhibiting renal glucose reabsorption and exert diverse cardiovascular protective effects [32,33,34]. SGLTs occur naturally in the mammalian brain, and SGLT2 inhibitors are lipid-soluble and can easily cross the blood–brain barrier [35,36]. SGLT2 inhibitors improved both glycemic control and cognitive function in patients with diabetes and animal models [37,38], likely owing to their anti-inflammatory and antioxidative properties, which also improve insulin resistance and peripheral glucose metabolism [39,40]. However, the effect of SGLT2 inhibitors on astrocyte-mediated hyperglycemia-induced inflammation remains unclear.

This study not only confirmed that EMPA effectively regulates hypothalamic inflammatory responses without changes in body weight by comparing short- and long-term HFD exposure but also highlighted the potent influence of SGLT2 inhibitors on inflammatory responses related to nutrient sensing in hypothalamic astrocytes. Additionally, we elucidated the specific mechanism by which SGLT2 inhibitors reduce the nuclear translocation of NF-κB and inhibit the expression of inflammatory cytokines, thereby establishing a direct link between SGLT2 inhibition and hypothalamic inflammation in metabolic diseases.

## 2. Materials and Methods

### 2.1. Animals

We sourced 8-week-old male C57BL/6N mice from Koatech (Namyangju-si, Republic of Korea) for the experiments, which were approved by the Institutional Animal Care and Use Committee of Pusan National University Yangsan Hospital (LT2023-007-A1C0). The mice were housed in a controlled environment with a 12 h light/dark cycle (lights on from 07:00 to 19:00) and a temperature of 23–25 °C. Mice were fed either a normal-fat diet (NFD, *n* = 5, 10% kcal fat) or HFD (*n* = 5, 60% kcal fat) for 3 d, 3 weeks, or 16 weeks. Subsequently, a 3-week EMPA treatment was administered (NFD or HFD + EMPA, *n* = 5, 10 mg/kg/d, oral gavage once a week for 3 weeks), and body weight and blood glucose levels were monitored.

### 2.2. Immunohistochemistry

The mice were anesthetized with tribromoethanol, transcardially perfused with ice-cold phosphate buffer (PB, 0.1 M, pH 7.4), and fixed with fresh 4% paraformaldehyde in PB. The fixed brains were post-fixed overnight at 4 °C, cut into 50-μm slices using a vibratome (VT1000P, Leica Microsystems, Wetzlar, Germany), and stored in PB. Coronal brain sections containing the hypothalamic arcuate nucleus were pre-incubated for permeabilization in 0.3% Triton X-100 (T8787, Sigma-Aldrich, St. Louis, MO, USA) in PB for 45 min, washed with PB, and incubated overnight at room temperature with a mouse anti-glial fibrillary acidic protein (GFAP) antibody (1:3000, G3893, Sigma-Aldrich). On the following day, the sections were washed with PB. For immunofluorescence staining, the sections were treated with goat anti-rabbit Alexa Fluor 594 (1:1000, A11012, Invitrogen, Carlsbad, CA, USA) for 2 h at room temperature. The stained brain sections were imaged using an FV-1200 confocal laser scanning microscope (Olympus America, Center Valley, PA, USA).

### 2.3. Cell Culture

Mouse astrocyte (C8-D1A) cells were maintained in Dulbecco’s modified Eagle’s medium (Welgene, Gyeongsan-si, Republic of Korea) supplemented with 10% heat-inactivated fetal bovine serum (Welgene) and 100 U/mL penicillin–streptomycin (Welgene) in a humidified atmosphere with 5% CO_2_ at 37 °C.

### 2.4. Cell Viability

The viability of the C8D1A cells was assessed using a 3-(4,5-dimethylthiazol-2-yl)-2,5-diphenyltetrazolium bromide (MTT) assay. C8D1A cells were seeded in 96-well plates (1 × 10^4^ cells/well; Corning, Corning, NY, USA) and incubated in a humidified atmosphere with 5% CO_2_ at 37 °C. The cells were then treated with various concentrations of EMPA (0, 0.5, 1, 10, 50, and 100 μM) or glucose (5.5, 17.5, 30, 55, and 100 mM) for 24 h. After treatment, 20 μL MTT (0.5 mg/mL; Merck KGaA, Darmstadt, Germany) was added to each well and incubated at 37 °C for 1 h. The resulting formazan crystals were dissolved in 200 μL dimethyl sulfoxide (Sigma Aldrich) at room temperature with agitation for 15 min. The absorbance was measured at 540 nm using a microplate spectrophotometer (BioTek Instruments, Winooski, VT, USA).

### 2.5. Immunocytochemistry

Mouse astrocyte C8D1A cells were washed with phosphate-buffered saline (PBS) and fixed in 4% paraformaldehyde at room temperature for 15 min. The fixed cells were incubated for 30 min at room temperature in a blocking solution containing 3% skim milk and 0.3% Triton X-100. After washing, the cells were treated with rabbit anti-NF-κB antibody (1:1000, SC-109, Santa Cruz Biotechnology, Dallas, TX, USA) overnight at 4 °C. The samples were incubated with an Alexa Fluor 488-labeled goat anti-rabbit (1:1000; A11008, Invitrogen) secondary antibody for 2 h at room temperature for immunofluorescence. Images were captured using an EVOS FL Auto2 Imaging System (Thermo Fisher Scientific, Waltham, MA, USA).

### 2.6. RNA Isolation and Quantitative Real-Time PCR

Total RNA was extracted from the hypothalamus and mouse astrocytic cell lines using the Sensi-TriJol reagent (Lugen SCI, Bucheon, Republic of Korea) according to the manufacturer’s instructions. For cDNA synthesis, 2 μg total RNA was reverse-transcribed using an UltraScript 2.0 cDNA Synthesis Kit (PCR Biosystems, London, UK). cDNA amplification was performed using real-time PCR with the following primer sets: GFAP sense, 5′-GAA-TGA CTC CTC CAC TCC CT-3′; GFAP antisense, 5′-ATG TAG CTA GCA AAG CGG TC-3′; IL-1β sense, 5′-AGG GCT GCT TCC AAA CCT TTG AC-3′; IL-1β antisense, 5 ′-ATA CTG CCT GCC TGA AGC TCT TGT-3′; TNF-α sense, 5 ′-TGG GAC AGT GAC CTG GAC TGT-3′; TNF-α antisense, 5′-TTC GGA AAG CCC ATT TGA GT-3′; β-actin sense, 5′-GGC TGT ATT CCC CTC CAT CG-3′; and β-actin antisense, 5′-CCA GTT GGT AAC AAT GCC ATG T-3′. Gene expression was normalized using β-actin as the internal control. A real-time PCR amplification of cDNA was performed using TOPreal SYBR Green qPCR PreMIX (Enzynomics, Daejeon, Korea) on a Light Cycler 96 (Roche Diagnostics, Rotkreuz, Switzerland) for around 40 cycles. Relative gene expression was calculated using the 2^−ΔΔCt^ method.

### 2.7. Cytosolic and Nuclear Fractionation

Mouse astrocyte C8D1A cells were washed twice with PBS and lysed on ice for 15 min with a hypotonic buffer [10 mM HEPES (pH 8.0), 60 mM KCl, 1 mM EDTA, and 0.075% (*v*/*v*) NP-40]. After centrifugation at 6000 rpm for 5 min, the supernatants were collected and used as the cytoplasmic fractions. Pellets were then lysed for 30 min on ice in hypertonic buffer [20 mM HEPES (pH 8.0), 0.2 mM EDTA, 25% (*v*/*v*) glycerol, 1.5 mM EDTA, and 420 mM NaCl] with brief vortexing. After centrifugation at 13,200 rpm for 10 min, the supernatants were collected and used as nuclear fractions. Extracted cytosolic and nuclear protein concentrations were obtained using a Bradford assay (Bio-Rad Laboratories, Hercules, CA, USA), and 15 μg of proteins from each sample were separated via SDS-PAGE and transferred to a PVDF membrane (Merck KGaA). The membranes were blocked with 5% non-fat skim milk in 0.1% TBST for 1 h at room temperature, followed by incubation at 4 °C with polyclonal antibodies against NF-κB (1:1000, SC-109, Santa Cruz Biotechnology), phospho-NF-κB (1:1000, sc-136548, Santa Cruz Biotechnology), Lamin B1 (1:2000, sc-377000, Santa Cruz Biotechnology), and β-actin (1:4000, sc-47778, Santa Cruz Biotechnology). After washing with 1× TBST, the membranes were incubated with horseradish peroxidase-conjugated mouse antibodies (1:3000, ADI-SAB-100, Enzo Biochem, New York, NY, USA) or rabbit secondary antibodies (1:3000, ADI-SAB-300, Enzo Biochem) for 2 h at room temperature. Immunoreactive signals were detected using Amersham ECL Prime Western Blotting Detection Reagent (GE Healthcare, Buckinghamshire, UK).

### 2.8. Statistical Analysis

Statistical analyses were performed using GraphPad Prism 10.2.1 (GraphPad Software, San Diego, CA, USA), with significance set at *p* < 0.05. Data were expressed as the mean ± standard deviation (SD). One-way or two-way analysis of variance was used for multiple group comparisons, along with Tukey’s multiple comparison tests for unequal replications. All data were confirmed to satisfy the normality assumption according to the Shapiro–Wilk test.

## 3. Results

### 3.1. Empagliflozin Reduces Weight Gain and Blood Glucose Levels in HFD-Induced Obese Mice

**Figure 1:**
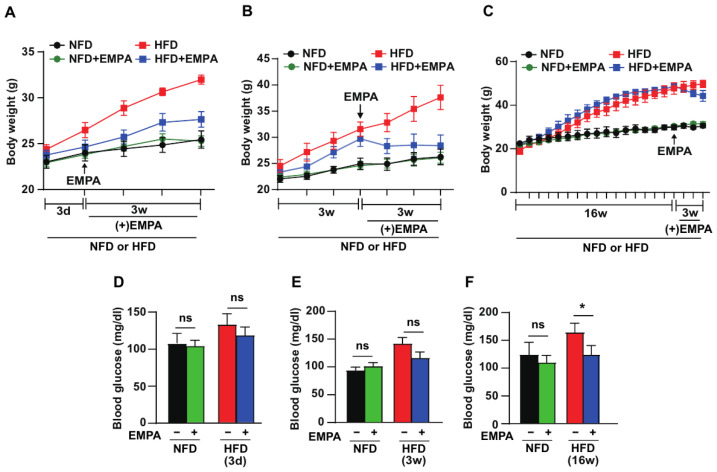
Effect of EMPA on body weight and blood glucose during HFD consumption. Body weight and blood glucose levels of mice treated with either vehicle or EMPA were measured after (**A**,**D**) 3 d, (**B**,**E**) 3 weeks, and (**C**,**F**) 16 weeks under NFD or HFD conditions, with EMPA administered for 3 weeks. *n* = 5 mice/group. Data are presented as the mean ± SD. * *p* < 0.05; ns, non-significant. EMPA: empagliflozin; NFD: normal-fat diet; HFD: high-fat diet.

We investigated the effects of EMPA on body weight (Figure 1A–C) and blood glucose levels (Figure 1D–F) in an 8-week-old mouse model of HFD-induced obesity. After 3 d of HFD feeding, body weight, and blood glucose levels did not change significantly. However, during the 3-week EMPA-treatment period, weight gain was lower in the HFD + EMPA group than in the HFD-only group (Figure 1A,D). In the 3-week HFD-fed group, body weight increased by ~10% compared to that in the NFD group. The HFD + EMPA group showed significantly lower weight gain and blood glucose levels than the HFD group (Figure 1B,E). In the 16-week HFD-fed group, body weight differences became more pronounced. EMPA treatment suppressed increases in both weight gain and blood glucose levels compared to the HFD (Figure 1C,F). These findings suggested that EMPA can regulate body weight and blood glucose levels in HFD-induced obesity.

### 3.2. Empagliflozin Reduces Hypothalamic Astrocyte Reactivation in HFD-Induced Obese Mice

**Figure 2:**
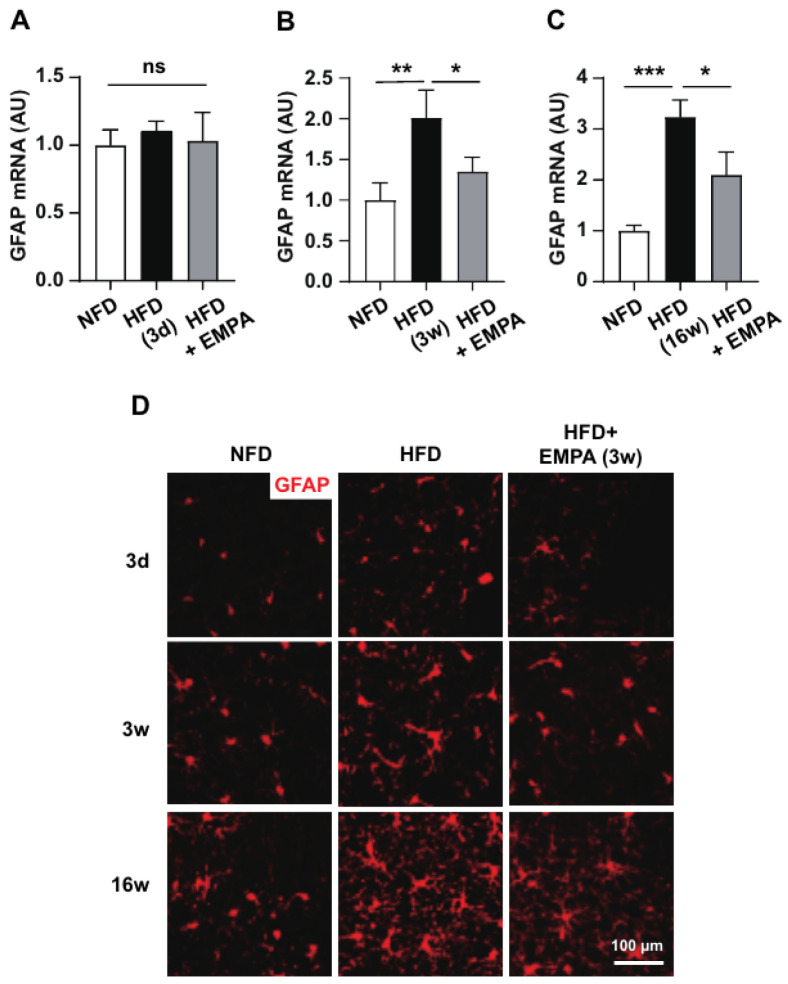
Effect of EMPA on astrocyte reactivation following HFD induction. (**A**) After 3 d, (**B**) 3 weeks, and (**C**) 16 weeks of the HFD, the hypothalamic mRNA expression of the astrocyte marker *GFAP* was assessed following 3 weeks of EMPA treatment. (**D**) Immunofluorescence analysis revealed that the number of reactive astrocytes in the hypothalamic arcuate nucleus was reduced in the HFD-fed groups following the EMPA treatment. Data are presented as the mean ± SD. * *p* < 0.05; ** *p* < 0.01; *** *p* < 0.001; ns, non-significant. Scale bar = 100 μm. GFAP: anti-glial fibrillary acidic protein.

We evaluated the effects of EMPA on astrocyte activation during HFD-induced CNS inflammation. We then evaluated the GFAP mRNA expression and histology of the reactive hypothalamic astrocytes. After 3 d of the HFD, EMPA treatment had no effects on reactive astrocytes or GFAP expression (Figure 2A,D). However, after 3 weeks of the HFD, hypothalamic GFAP expression increased and was significant after EMPA treatment, along with the number of reactive astrocytes (Figure 2B,D). After 16 weeks of the HFD, mice exhibited a >50% increase in body weight and substantial increase in reactive astrocyte numbers, which were rescued by the 3-week EMPA treatment (Figure 2C,D). These findings suggested that EMPA participates in astrocyte activation during HFD-induced hypothalamic inflammatory responses.

### 3.3. Empagliflozin Regulates Hypothalamic Inflammatory Responses in HFD-Induced Obese Mice

**Figure 3:**
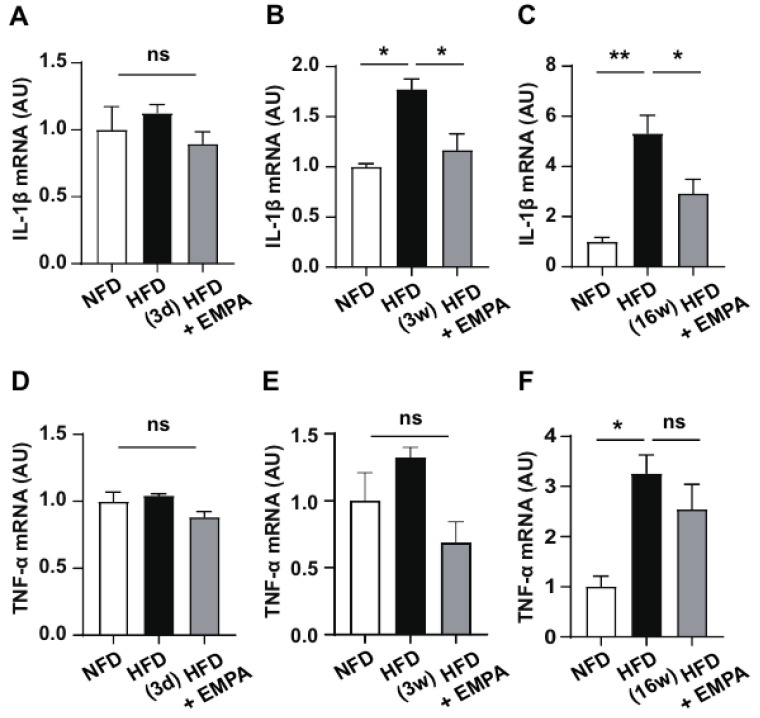
Effects of EMPA on cytokine expression in hypothalamic inflammatory responses induced by HFD. mRNA levels of *IL-1β* (**A**–**C**) and *TNF-α* (**D**–**F**) detected by real-time PCR. Data are presented as the mean ± SD. * *p* < 0.05; ** *p* < 0.01; ns, non-significant.

HFD-induced hypothalamic inflammation is closely linked to metabolic disorders such as obesity. We evaluated the anti-inflammatory effects of EMPA in the hypothalamus of HFD-induced obese mice based on the changes in the mRNA levels of pro-inflammatory cytokines, including *TNF-α* and *IL-1β*. Mice fed the 3 d HFD showed no significant changes in cytokine expression, limiting the potential effect of EMPA (Figure 3A,D). Mice fed the 3-week HFD showed increased levels of *IL-1β* mRNA, which was significantly reduced by the EMPA treatment (Figure 3B). *TNF-α* expression was also significantly decreased by EMPA (Figure 3E). Mice fed the 16-week HFD showed increased levels of both *IL-1β* and *TNF-α*, and EMPA treatment significantly reduced *IL-1β* expression (Figure 3C,F). These results suggest that EMPA is closely associated with inflammatory responses through the regulation of cytokine expression in the hypothalamus.

### 3.4. Empagliflozin Regulates NF-κB Signaling in Astrocytes Under Hyperglycemic Conditions

**Figure 4:**
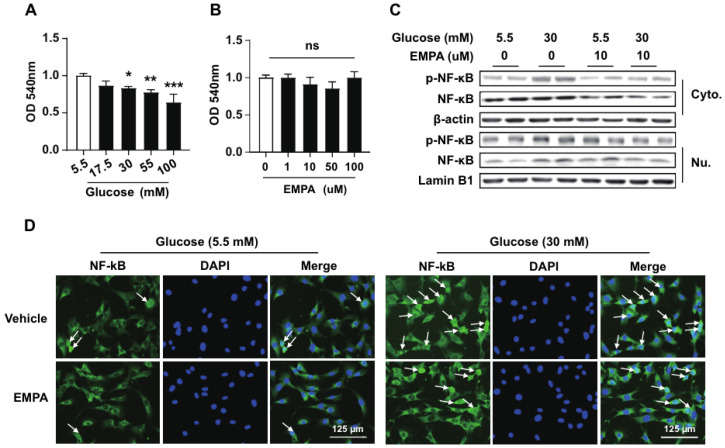
Effects of EMPA on NF-κB P65 expression in astrocytes. (**A**,**B**) Viability of astrocyte cell line C8D1A assessed using MTT assay. Cells were treated with varying concentrations of glucose (5.5–100 mM) and EMPA (0–100 μM) for 24 h. (**C**) Immunoblotting results show the expression of NF-κB P65 in the cytoplasm and nucleus under high concentrations of glucose and EMPA. (**D**) Immunocytochemical images demonstrating the expression and nuclear translocation of NF-κB (arrows). Green fluorescence indicates the localization of NF-κBsubunits, while blue fluorescence represents nuclear DAPI staining. Data are presented as the mean ± SD. * *p* < 0.05; ** *p* < 0.01 *** *p* < 0.001; ns, non-significant. Scale bar = 125 μm.

Given the important regulatory role of NF-κB signaling in inflammatory pathways, we evaluated the regulation of P65 phosphorylation, a key protein in the NF-κB signaling cascade, by EMPA in mouse astrocyte C8D1A cells using immunoblotting. The MTT assay showed that increasing concentrations of glucose reduced cell viability (Figure 4A), and EMPA had minimal impact on cell viability, even at high concentrations (Figure 4B). The levels of P65 phosphorylation were significantly elevated under high glucose levels but were suppressed by EMPA treatment. Moreover, high glucose levels promoted the nuclear translocation of cytosolic NF-κB P65, which was partially inhibited by EMPA (Figure 4C). Immunofluorescence analysis confirmed that the number of p-P65-positive cells in astrocyte nuclei significantly increased after high-glucose treatment and that EMPA reduced the glucose-induced nuclear translocation of P65 (Figure 4D). These findings suggested that EMPA is a potent anti-inflammatory modulator in astrocyte-mediated inflammatory responses under hyperglycemic conditions.

## 4. Discussion

Recent studies on obesity have reported that hypothalamic inflammation induced by an energy-excessive state leads to hypothalamic dysfunction [41]. This study examined the response of astrocytes to the SGLT2 inhibitor EMPA in the context of HFD-induced hypothalamic inflammation and demonstrated its important anti-inflammatory role in the CNS. These findings highlight a novel function of EMPA in the modulation of hypothalamic inflammation via the regulation of astrocyte activation. Importantly, the hypothalamic inflammatory response induced by EMPA may influence inflammation in models of short-term HFD consumption, regardless of changes in body weight. Recent studies have shown that HFD-induced obesity increases the biochemical molecules that trigger metabolic disorders, resulting in inflammation within the CNS, including the hypothalamus. This inflammatory response disrupts insulin and leptin signaling and affects neuronal function through the accumulation and activation of astrocytes—both important contributors to the onset and progression of various metabolic diseases [42,43,44]. The interactions between non-neuronal and neuronal cells are closely associated with central inflammatory pathologies. As key modulators of neuroinflammation, astrocytes are crucial in neurotransmission by supplying energy to neurons and releasing neurotransmitters [45,46]. However, the chronic activation of astrocytes induced by HFD-related obesity can increase cytokine expression and reduce leptin signaling sensitivity in hypothalamic neurons [47,48]. The overactivation of astrocytes is thought to directly contribute to brain inflammation associated with both physiological responses and pathological states [49]. Given that hypothalamic inflammation is strongly linked to disturbances in energy homeostasis [50,51], we hypothesized that EMPA actively participates in reducing hypothalamic inflammation.

This study investigated the regulatory effects of EMPA on reactive astrocytes (as indicated by GFAP expression) and inflammatory cytokines *(TNF-α* and *IL-1β*) in HFD-induced hypothalamic inflammation. By comparing short- and long-term HFD exposures, we confirmed that EMPA effectively regulates hypothalamic inflammatory responses, even without weight changes during short-term HFDs. An HFD increases the expression of cytokines through various mechanisms, regardless of weight change, leading to inflammation. Circulating inflammatory cytokines and FFAs rapidly induce inflammation in the hypothalamus, particularly in the median eminence, which lacks a blood–brain barrier [52]. Glial cell activation due to inflammatory cytokine (e.g., *TNF-α* and *IL-1β*) accumulation leads to gliosis [41,53], accompanied by inflammation-related tissue damage [41,54]. During hypothalamic inflammation, astrocytes accumulate lipid droplets under high FFA levels [55,56], leading to activation and structural changes [57,58]. Recent studies have indicated that SGLT2 inhibitors effectively regulate cytokine secretion in cardiovascular diseases [59]. Furthermore, SGLT2 inhibitors alleviated nephrotic and hepatic inflammation via NF-κB signaling pathways [60,61].

Consistent with the findings of numerous studies, we demonstrated that EMPA effectively regulates blood glucose levels and inhibits the hypothalamic expression of pro-inflammatory cytokines to modulate immune responses under an experimentally induced metabolic disease state. In contrast to previous studies that primarily utilized diabetic animal models, our study utilized an HFD-induced animal model, provides new insights into inflammation related to obesity, and emphasizes the specific mechanisms by which EMPA influences hypothalamic inflammatory responses.

### Study Limitations and Future Perspectives

Chronic HFD consumption has been reported to activate the inflammatory NF-κB signaling pathway. Previous studies have indicated that selectively inhibiting NF-κB function in neurons or glial cells reduces astrocyte activation and promotes weight loss [62]. In this study, EMPA suppressed astrocyte activation following HFD consumption, as evidenced by the changes in the number and morphology of GFAP-stained cells in the hypothalamus. Immunoblotting and immunofluorescence analysis indicated that SGLT2 inhibition inhibited the nuclear translocation of P65 in astrocytes under hyperglycemic conditions, thereby diminishing NF-κB pathway activation and inhibiting inflammatory cytokine expression. The current study only presents evidence on the inhibition of NF-κB signaling in astrocyte cultures; thus, further research is needed to explore the specific functions of glial cells in animal models. The saturated fatty acids in an HFD promote astrocyte activation in the hypothalamus, resulting in neuronal damage, particularly in regions involved in energy homeostasis. Recent studies demonstrated that astrocytes disrupt nutrient sensing in the hypothalamus. Inhibiting astrocyte activation via NF-κB-dependent mechanisms can reduce food cravings and alleviate diet-induced obesity. These findings suggest that the SGLT2 inhibitor EMPA decreases astrocytic inflammatory responsiveness, thereby inhibiting inflammatory mediator secretion and hypothalamic inflammation under HFD conditions. More comprehensive studies are needed on the mechanisms by which EMPA rescues the metabolic disease and inflammation profiles. They should explore interactions with additional signaling pathways to better understand its full scope of action. Comparing the effects of EMPA and other SGLT2 inhibitors will help to clarify the specific mechanisms and clinical advantages of each drug. Research into the neuroprotective and metabolic roles of EMPA during long-term use is crucial for demonstrating its safety and efficacy. SGLT2 inhibitors are promising therapeutic agents against hypothalamic inflammation and related metabolic and neurodegenerative diseases associated with HFDs.

## 5. Conclusions

EMPA inhibited the nuclear translocation of NF-κB under hyperglycemic conditions, thereby reducing the expression of pro-inflammatory cytokines, such as *TNF-α* and *IL-1β*, and inhibiting astrocyte reactivation. This regulatory mechanism may be crucial in modulating hyperglycemia-induced inflammation in the CNS.

## Data Availability

Data are contained within the article.
